# Components of a Mediterranean diet and their impact on cognitive functions in aging

**DOI:** 10.3389/fnagi.2015.00132

**Published:** 2015-07-08

**Authors:** Sebastian Huhn, Shahrzad Kharabian Masouleh, Michael Stumvoll, Arno Villringer, A. Veronica Witte

**Affiliations:** ^1^Department of Neurology, Max Planck Institute for Human Cognitive and Brain SciencesLeipzig, Germany; ^2^Collaborative Research Centre 1052 ‘Obesity Mechanisms’, Subproject A1, Faculty of Medicine, University of LeipzigLeipzig, Germany; ^3^IFB Adiposity Diseases, University of LeipzigLeipzig, Germany

**Keywords:** cognition, plasticity, omega-3 fatty acids, polyphenols, resveratrol, memory, brain structure

## Abstract

**Background**: Adhering to the Mediterranean diet (MeDi) is known to be beneficial with regard to many age-associated diseases including cardiovascular diseases and type 2 diabetes. Recent studies also suggest an impact on cognition and brain structure, and increasing effort is made to track effects down to single nutrients.

**Aims**: We aimed to review whether two MeDi components, i.e., long-chain omega-3 fatty acids (LC-n3-FA) derived from sea-fish, and plant polyphenols including resveratrol (RSV), exert positive effects on brain health in aging.

**Content**: We summarized health benefits associated with the MeDi and evaluated available studies on the effect of (1) fish-consumption and LC-n3-FA supplementation as well as (2) diet-derived or supplementary polyphenols such as RSV, on cognitive performance and brain structure in animal models and human studies. Also, we discussed possible underlying mechanisms.

**Conclusion**: A majority of available studies suggest that consumption of LC-n3-FA with fish or fishoil-supplements exerts positive effects on brain health and cognition in older humans. However, more large-scale randomized controlled trials are needed to draw definite recommendations. Considering polyphenols and RSV, only few controlled studies are available to date, yet the evidence based on animal research and first interventional human trials is promising and warrants further investigation. In addition, the concept of food synergy within the MeDi encourages future trials that evaluate the impact of comprehensive lifestyle patterns to help maintaining cognitive functions into old age.

## Background: Health Benefits of the Mediterranean Diet

According to the “Global Strategy on Diet, Physical Activity and Health”, a review developed by the World Health Organization (WHO), the Mediterranean Diet (MeDi) is a promising strategy to prevent from diseases and enhance quality of life (World Health Organization, 2009). The review aims specifically on interventions, that reduce the risk for non-communicable diseases like cerebro- and cardiovascular diseases, cancer, respiratory diseases, diabetes and neurodegenerative diseases, which comprise the leading causes of death worldwide (World Health Organization, 2009). The MeDi was first investigated by Ancel Keys in the 1950s during his Seven Countries Study, a large-scale prospective cohort-study with more than 11,000 participants (Keys, 1970; Keys et al., 1986). Keys et al. (1986) observed a considerable difference in the eating pattern of Southern European countries, compared to Northern Europe and the USA. This Mediterranean eating pattern and related low intake percentage of total energy from saturated fatty acids correlated with lower serum cholesterol and lower blood pressure in Mediterranean countries, which were again associated with a lower coronary mortality and a lower risk for the above mentioned diseases in comparison to countries adhering to a Western-type diet (Keys et al., 1986).

Distinctive for the MeDi is the high consumption of fruits, vegetables, grains as well as sea-fish on regular basis, while the intake of meat and dairy products, just as sweets and convenience food is rather low (Trichopoulou et al., 1995; Gotsis et al., 2015). In addition, the regular consumption of red wine (mainly served with food) and olive oil (as principal source of fat) is characteristic for the MeDi (Willett et al., 1995). For a detailed description of the MeDi, often displayed as food pyramid, see Bach-Faig et al. (2011).

Over the last decades, epidemiologic studies supported and extended Keys’ findings to a multitude of health benefits that are provided by the MeDi, e.g., with regard to cancer and cardiovascular diseases (Couto et al., 2011; Lopez-Garcia et al., 2014; Gotsis et al., 2015). More recently, research also focused on neurodegenerative diseases and the impact of MeDi on cognition. For reviews, see e.g., Lourida et al. (2013) and van de Rest et al. (2015). For example, Scarmeas et al. (2006) observed in a prospective cohort of 2258 community-based non-demented individuals that higher adherence to the MeDi is associated with a significant reduction in the risk for Alzheimer’s disease (AD). In a systematic review, Lourida et al. (2013) described a reasonably consistent pattern of associations between adherence to the MeDi and related lower risks for AD, reduced rates of cognitive decline as well as better cognitive function. Most recently, Valls-Pedret et al. (2015) described positive results of a long-term randomized clinical trial (RCT) in 334 participants with high cardiovascular risk at a mean age of 67 years (PREDIMED study), providing an even stronger level of scientific evidence than results based on observational studies (Valls-Pedret and Ros, 2013): Here, a MeDi supplemented with either olive oil or nuts, in comparison to a control diet, was associated with improved cognitive functions at 4-year follow-up (Valls-Pedret et al., 2015).

These beneficial effects might be due to multiple biological mechanisms, such as lower concentrations of serum-cholesterol in Mediterranean areas and a related decrease of cardiovascular risk, which were among the first findings by Keys et al. (1986). More specifically, adherence to the MeDi is associated with a reduced risk for coronary heart diseases and metabolic syndrome including hypertension and dyslipidemia, which have been associated with the development of cognitive impairments (for review see e.g., van den Berg et al., 2009; Yates et al., 2012). Additionally, adhering to the MeDi might prevent from disturbances in insulin/glucose metabolism that can result in type 2-diabetes mellitus (DM-2), which is associated with an increased risk for AD and cognitive impairments (Biessels et al., 2006; Hu et al., 2013). Even in the absence of manifest DM-2, chronically elevated levels of blood-glucose have shown to exert negative effects on AD risk and memory performance in older adults (Crane et al., 2013; Kerti et al., 2013).

In sum, the MeDi has been shown to exert positive effects on risk for AD and cognitive functions during aging, which is probably mediated through reductions in vascular risk factors and benefits on lipid and glucose metabolism. Moreover, based on animal research it has been postulated that specific nutrients could exert even more directly protective effects on the aging brain, e.g., considering amyloid-beta metabolism (Allès et al., 2012). As the MeDi is a complex eating pattern, though, a multitude of single components could cause beneficial effects (Jacobs et al., 2009; Gotsis et al., 2015). Understanding these underlying mechanisms and eventually develop preventive and therapeutic strategies based on those insights, are important issues for future research.

This review aims to evaluate recent findings concerning the effects of single components of the MeDi and their impact on cognition. Firstly, we focus on long chain omega-3 fatty acids (LC-n3-FA) derived from fish, as they distinguish the MeDi from other diets and are consumed with high frequency (Tangney et al., 2014). Secondly, our focus is on plant polyphenols (including resveratrol), which occur mainly in fruit, tea and red wine (Manach et al., 2004). The deliberate consumption of red wine is a well-known feature of the MeDi and especially resveratrol is assigned beneficial effects with regard to overall health, as well as cognition (Baur and Sinclair, 2006; Witte et al., 2014). Both nutrients attracted increasing research interest in the last years.

## Impact of Omega-3 Fatty Acids on the Brain

**Figure 1:**
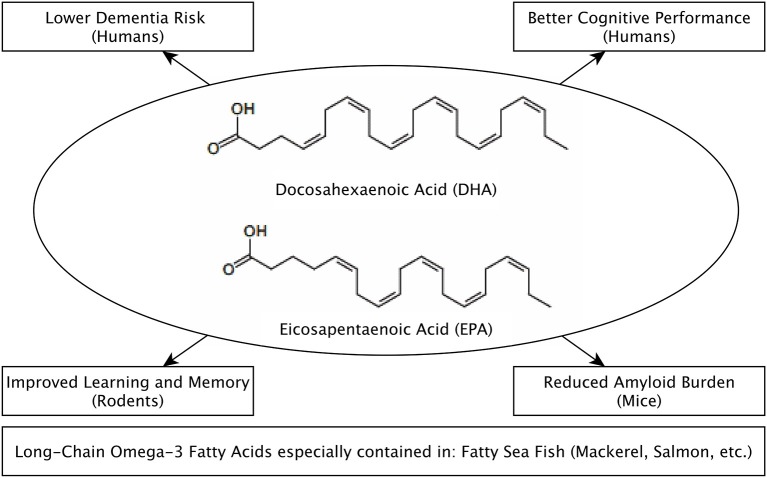
**Postulated effects of long-chain Omega-3 Fatty Acids (Eicosapentaenoic Acid, EPA and Docosahexaenoic Acid, DHA) with regard to brain health and their main dietary sources**.

One characteristic of the MeDi is a high intake of unsaturated fatty acids, including the long-chain omega-3 polyunsaturated fatty acids (LC-n3-FA) eicosapentaenoic acid (EPA, C20:5, n-3) and docosahexaenoic acid (DHA, C22:6, n-3; Figure 1). The main source of DHA and EPA in the human diet is fatty sea fish like mackerels or salmon (Max Rubner-Institut, 2011). DHA and EPA cannot be efficiently synthesized by human enzymes and are therefore regarded as semi-essential (Burdge and Calder, 2005; Burdge, 2006; Sala-Vila and Ros, 2011). Astrocytes in the brain are a major site for the processing of LC-n3-FA. They elongate and desaturate precursor fatty acids such as linoleic acid and the vegetable LC-n3-FA alpha-linolenic acid (ALA) to form EPA and DHA (Moore et al., 1991). Notably, not only the absolute amount of DHA and EPA might be important, but also the ratio of the precursors, as with different precursor ratios, different conversion rates to DHA and EPA occur (Kaur et al., 2014). In addition, intake of ALA, contained e.g., in nuts, might also directly contribute to the beneficial effects of the MeDi on cognition (Blondeau et al., 2009; Valls-Pedret et al., 2015; for a detailed discussion of possibly distinct effects of ALA, EPA and DHA please see Freemantle et al., 2006).

It is widely accepted, that LC-n3-FA are crucial for the growth and development of the infant brain during pregnancy and after birth (Kris-Etherton et al., 2009). The predominant LC-n3-FA DHA alone comprises 10–20% of total fatty acids of the brain and is thought to be important with regard to neuronal differentiation, synaptogenesis, and synaptic function (McNamara and Carlson, 2006). It has also been proposed that the access to DHA during hominid evolution played a key role in increasing the brain to body-mass ratio (Crawford et al., 1999, 2013). However, due to the easy availability of processed food in Western societies today, the consumption of saturated fatty acids and trans-fatty acids increased, while that of DHA decreased. This has been speculated to contribute to an increased incidence of brain disorders such as major depression (Su et al., 2003).

Considering the abundance of DHA in brain tissue and its importance for brain development and evolution, it is reasonable to suppose that DHA also contributes to the evolvement and maintenance of proper cognitive functioning in later life (Gómez-Pinilla, 2008). Indeed, several experimental animal studies demonstrated superior learning and better memory performance in rodents that received supplementary DHA with their diet (Morris et al., 2005). DHA might have a beneficial impact even during pathological conditions like AD. Lim et al. (2005) found in aged mice on a DHA-enriched diet a significant reduction of total amyloid β (A-β) by more than 70% when compared with low-DHA or control chow diets. This could be neuroprotective, given the probable downstream toxicity of A-β deposition and its implications in the development of AD (Lim et al., 2005). That is a further finding on the protective properties of DHA against synaptic loss, which is a critical issue in concerns of AD and seems to support the hypothesis that DHA is protective against AD (Calon et al., 2004).

These findings are in line with human epidemiological studies that report associations between the consumption of fish in general (Barberger-Gateau et al., 2005; Morris et al., 2005), as well as LC-n3-FA e.g., as dietary fishoil supplement (McCann and Ames, 2005; Gómez-Pinilla, 2008), with better cognitive performances and lower risk of dementia (for a review, see Fotuhi et al., 2009). For example, a large-scale prospective cohort study with 6158 residents of a community in Chicago of 65 years and older, estimated that fish consumption was associated with slower cognitive decline with age, assessed using a global cognitive score (Morris et al., 2005).

The evidence for positive effects of LC-n3-FA fishoil supplementation on cognitive functions in normal and pathological aging based on placebo-controlled RCTs is less clear, see Table 1 for an overview. In an early double-blind RCT in 204 AD patients, Freund-Levi et al. (2006) observed positive effects of LC-n3-FA in a small group of those with very mild AD who took supplementary LC-n3-FA over 6 months. These findings are in line with a 24-week RCT by Chiu et al. (2008) in 46 participants. Here the authors also concluded that LC-n3-FA improved general clinical function in patients with mild or moderate AD, as well as mild cognitive impairment (Chiu et al., 2008). In an own double-blind prospective interventional study, it was shown that LC-n3-FA improved executive functions and gray matter volume, as well as white matter microstructure in healthy older individuals, after 26 weeks of fish oil supplementation (Witte et al., 2013). Yurko-Mauro et al. (2010) observed in another RCT with 485 healthy subjects older than 55 years that 24 weeks of supplementation with 900 mg/d DHA improved learning and memory function.

**Table 1:** **Characteristics of studies reporting associations between fish-consumption or LC-n3-FA-supplementation and cognition**.

Author (year)	Participants		Duration	Intervention		Measured outcome		Results	
	sample size/age (years)	
Chiu et al. (2008)	*N* = 46 memory complaints	I: 74.0 (70.1–77.8) P: 76.5 (71.8–81.1)	24 weeks	1.8 g Omega-3 PUFAs/d	Placebo	ADAS-cog		AD group: ○	MCI group: +
Dangour et al. (2010)	*N* = 867 healthy	I: 74.7 ± 2.5 P: 74.6 ± 2.7	24 months	200 mg EPA + 500 mg DHA/d	Placebo	Extensive NP test battery		Whole group: ○	
Freund-Levi et al. (2006)	*N* = 204 AD	I: 72.6 ± 9.0 P: 72.9 ± 8.6	6 months	1.7 g DHA/d and 0.6 g EPA/d	Placebo	ADAS-cog	MMSE	Whole group: ○	Sub- group: +
Morris et al. (2005)	*N* = 6185 healthy	I1: 74.6 I2: 74.2 I3: 73.9	6 years	Observational		Global cognitive score		Whole group: +	
Quinn et al. (2010)	*N* = 402 mild to moderate AD	I: 76 ± 9.3 P: 76 ± 7.8	18 months	2 g/d DHA	Placebo	ADAS-cog	Clinical Dementia Rating (CDR) sum of boxes	Whole group: ○	
Reddy et al. (2011)	*N* = 27 schizophrenia	18–45	24 weeks	2 g/d EPA		Wisconsin Card Sort Test		Whole group: +	
Tan et al. (2012)	*N* = 1575 healthy	67 ± 9	–	Observational (Red blood cell EPA + DHA)		Extensive NP test battery		Whole group: +	
van de Rest et al. (2008)	*N* = 302 healthy	I1800: 69.9 ± 3.4 I400: 69.5 ± 3.2 P: 70.1 ± 3.7	26 weeks	1800 mg/d EPA-DHA 400 mg/d EPA-DHA	Placebo	Extensive NP test battery		Whole group: ○	
Witte et al. (2013)	*N* = 65 healthy	I: 65 ± 6.3 P: 62.9 ± 6.8	26 weeks	2.2 g/d EPA-DHA	Placebo	Extensive NP test battery		Whole group: +	
Yurko-Mauro et al. (2010)	*N* = 485 healthy	I: 70 ± 9.3 P: 70 ± 8.7	24 weeks	900 mg DHA/d		CANTAB Paired Associate Learning		Whole group: +	

*AD, Alzheimer’s Disease; ADAS-cog, Alzheimer’s Disease Assessment Scale, cognitive subscale; DHA, docosahexaenoic acid; EPA, eicosapentaenoic acid; I, age of intervention group; MCI, Mild Cognitive Impairment; MMSE, Mini-Mental State Examination; NP, neuropsychological; P, age of placebo group; +, positive effect on cognition; ○, no effect on cognition*.

Supporting these findings, Pottala et al. (2014) observed in a cross-sectional analysis, that a higher LC-n3-FA intake (indicated by higher proportions of DHA and EPA in the membranes of blood erythrocytes, see Harris and Von Schacky, 2004) was correlated to higher total brain and hippocampal volume in 1111 postmenopausal women. In another cross-sectional study by Tan et al. (2012) in 1575 elderly participants, those with lower DHA had also lower scores on tests of executive function and abstract thinking. Similarly, executive functions could be improved after 24 weeks of supplementary LC-n3-FA intake (2 g EPA/d) in 27 schizophrenic patients in an open-label study (Reddy et al., 2011).

In contrast, other interventional studies in AD patients (Quinn et al., 2010) or healthy older adults (van de Rest et al., 2008; Dangour et al., 2010) did not support the positive effects of fish oil consumption. These inconsistent results might be explained due to differences in dosage and duration between studies, e.g., that LC-n3-FA intake might not have been sufficient to exert statistically significant effects on cognition. Furthermore studies might differ in intake instructions and cohort characteristics. It has also been noted that not only the amount of LC-n3-FA, but also the overall dietary fat-composition is considerably critical for brain functions (Morris et al., 2005). For example, an unfavorable fat composition might affect cognitive aging more than total fat intake itself (Okereke et al., 2012). Especially saturated fatty acids and trans-fatty acids are supposed to increase the risk of AD (Hooijmans et al., 2007; Studzinski et al., 2009; Ramassamy and Belkacémi, 2011) and affect cognition (Greenwood and Winocur, 2005), which could be due to decreased Brain-derived neurotrophic factor (BDNF) related synaptic plasticity (Molteni et al., 2002). Thus, it might be speculated that the positive effects of supplementary LC-n3-FA could be masked out by the negative effects of concurrent high saturated- and trans- fatty acid intake. According to the latest Cochrane reviews, it is not yet clear that dietary or supplemental LC-n3-FA alter total mortality, combined cardiovascular events or cancers in people with, or at high risk of, cardiovascular disease or in the general population (Hooper et al., 2004). The same stated Sydenham et al. (2012) for LC-n3-FA and dementia. They could not state benefits for cognitive health for older people taking omega-3 supplements. However, none of the mentioned studies reported severe adverse effects of fish or fish oil consumption.

Underlying mechanisms of positive effects of LC-n3-FA on cognition could include a reduction of cardiovascular risk factors, e.g., by improving cerebral blood flow and lowering triacylglycerol levels as found in non-human primates and rats (Katayama et al., 1997; Tsukada et al., 2000; Fotuhi et al., 2009). More direct neuronal effects of LC-n3-FA are e.g., stimulation of neurogenesis and neurite outgrowth (Kawakita et al., 2006) and enhancement of synaptic membrane fluidity (Cansev and Wurtman, 2007). Also, LC-n3-FA have been found to increase the expression of myelin-related proteins (Salvati et al., 2008), which could contribute to improved axonal transmission and thus better neuronal signaling. In addition, LC-n3-FA are thought to upregulate several genes such as Sir2, involved in maintaining synaptic function and plasticity (Wu et al., 2007). A recent study in mice showed an increase of neuroprotectin D-1 (NPD-1) after fish oil treatment (Afshordel et al., 2015). NPD-1 represents a neuroprotective compound that is derived from unesterified DHA (Afshordel et al., 2015).

Moreover, LC-n3-FA play several roles with regard to inflammatory processes. DHA and EPA are capable of competing with arachidonic acid in the production of eicosanoids, which results in the production of biologically less active thromboxans and therefore in a better hemodynamic, vascular tone and inflammation (Mori and Beilin, 2004). LC-n3-FA might also upregulate the expression of antioxidant enzymes and downregulate genes associated with production of reactive oxygen species (ROS), such as peroxisome proliferator-activated receptors gamma (PPAR-γ; Takahashi et al., 2002; Mori and Beilin, 2004). Additionally, DHA has been implicated in reducing inflammation through fatty acid derivatives such as NPD-1 (Cole et al., 2010) and resolvin species (Kohli and Levy, 2009).

In sum, promising evidence indicates that LC-n3-FA, especially DHA, exert positive effects on brain structure and cognitive functions. Yet, more large-scale RCTs are needed before fish oil intake could be fully recommended as preventive strategy against cognitive decline in the older population.

## Polyphenols and their Impact on the Brain

**Figure 2:**
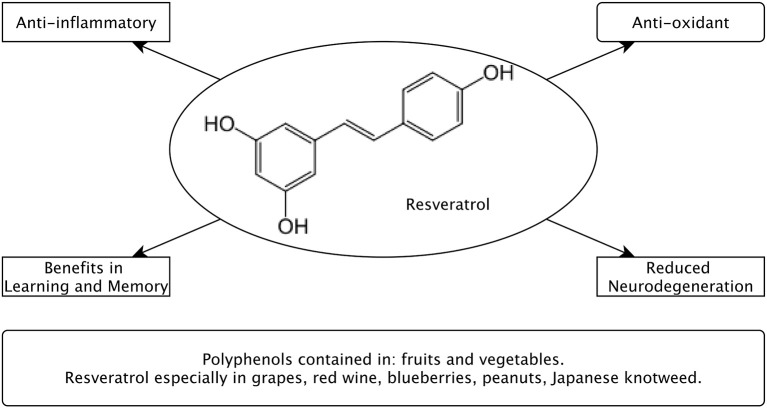
**Postulated effects of Polyphenols including Resveratrol with regard to brain health and their main dietary sources**.

A further class of substances that is supposed to contribute to the beneficial effects of the Mediterranean Diet (MeDi) is that of polyphenols (Figure 2). Polyphenols are secondary metabolites of plants and characterized by the chemical structure of hydroxyl groups on aromatic rings (Manach et al., 2004). They are quite abundant in our diet and several thousand molecules have been identified to have polyphenol character (Manach et al., 2004). One polyphenol agent that came into research focus is resveratrol (RSV). It occurs naturally in the skin of red grapes, red wine, blueberries, peanuts and Japanese knotweed (Baur and Sinclair, 2006; Baur et al., 2006; Ingram et al., 2006). Another group, the flavonols, are part of the flavonoid family that is found in various fruits, cocoa, beans and the Ginkgo biloba tree (Gómez-Pinilla, 2008). Flavonols contain anti-inflammatory properties among several other complex actions (for review, see Gómez-Pinilla, 2008). Although polyphenols are somewhat heterogeneous regarding their chemical properties, they seem to have some effects in common with regard to cardiovascular health and (at least for some polyphenols) antioxidant capacity (Halliwell, 2007; Habauzit and Morand, 2012).

*In vitro*, several polyphenols including RSV eliminate a multitude of ROS, including hydroxyl radicals, peroxyl radicals hypochlorous acid and in part superoxide radical (Halliwell, 2007). ROS are considered to be toxic and ROS-induced cell damage is assumed to contribute to the process of aging (Liochev, 2013). In rats, polyphenols have been shown to increase heat-shock protein (HSP) 70 and insulin-like growth factor 1 (IGF-1) expression in the hippocampus, which protects against kainate-induced cell damage and benefits learning and memory performance (Casadesus et al., 2004; Galli et al., 2006). Reduced hippocampal neurodegeneration has also been shown after RSV administration in rodent models for AD/tauopathies (Kim et al., 2007). In addition, administration of RSV-containing red wine was found to preserve spatial memory, while reducing Aβ neuropathology (Wang et al., 2010). In a non-human primate study, supplementary RSV for 18 months increased spatial memory performance compared to placebo (Dal-Pan et al., 2011).

Considering human studies, there is a considerable heterogeneity in study quality, design and polyphenol formula/dosage (Crichton et al., 2013). See Table 2 for an overview. In a cross-sectional study by Nurk et al. (2009) with 2,031 participants aged 70–74 years from the Hordaland Health Study in Norway, a diet over 1 year high in some flavonol-rich foods, such as chocolate, wine and tea, was associated with better performance in several cognitive abilities in a dose-dependent manner in comparison to a non-consumer group. Only a few placebo-controlled interventional studies are available to date, such as Kennedy et al. (2010). This study assessed the effects of 250 and 500 mg oral RSV on cognitive performance in a RCT crossover study in 22 healthy adults, with the result that even single doses of orally administered RSV can modulate cerebral blood flow variables, measured using MRI (Kennedy et al., 2010). In another study, blueberry supplementation (wild blueberry juice) improved paired associate learning and word list recall, as well as paired associate learning in a small sample of nine older adults after comparison with a matched, placebo-controlled sample (Krikorian et al., 2010). In a double-blind, clinical trial by Small et al. (2014) intake of a pill-based nutraceutical that contained a proprietary formulation of blueberry (including RSV), green tea, carnosine, vitamin D3 and biovin, resulted in significantly increased processing speed of 52 participants compared to placebo (*N* = 53). In an own study with 46 healthy overweight older individuals, a daily intake of 200 mg RSV (in a formula with quercetin) over 26 weeks compared to placebo intake significantly improved memory performance (Witte et al., 2014). In addition, glycated hemoglobin (HbA1c) in peripheral blood was significantly reduced after RSV treatment, and this reduction in HbA1c correlated with higher functional connectivity of the hippocampus, measured using resting-state functional MRI in the same subjects. Notably, changes in functional connectivity were found to correlate with the observed increases in memory, pointing to ameliorated glucose metabolism as one underlying mechanism of the positive effects of RSV on cognition (Witte et al., 2014). Also, Brickman et al. (2014) reported recently in a randomized study on flavonols with 37 healthy 50 69 year old subjects using functional MRI that a diet high in cocoa-flavanol over 3 months enhanced memory function and improved related activation in the dentate gyrus, the hippocampus region characterized by life-long neurogenesis, in comparison to a diet low in cocoa-flavanol.

**Table 2:** **Characteristics of studies reporting associations between flavonol or RSV consumption and cognition**.

Author (year)	Participants		Duration	Intervention			Measured outcome		Results (Polyphenol)
	sample size/age	
Kennedy et al. (2010)	*N* = 22	Healthy 20.17 y	Single dose	250 mg (RSV)	500 mg (RSV)	Placebo	Cognitive task	Cerebral blood flow	+
Krikorian et al. (2010)	*N* = 9, placebo *N* = 7	Healthy 76.2 ± 5.2 y	12 weeks	Daily consumption of wild blueberry juice			Paired associate learning	Word list recall	+
Nurk et al. (2009)	*N* = 2031	Healthy 70–74 y	Cross-sectional	Observational (Chocolate, Wine, Tea)			Extensive NP test battery		+
Small et al. (2014)	*N* = 52, placebo *N* = 53	Healthy I: 72.82 P: 74.34	2 months	Pill-based nutraceutical		Placebo	Extensive NP test battery		+
Witte et al. (2014)	*N* = 23, Placebo *N* = 23	Healthy, overweight I: 64.8 ± 6.8 P: 63.7 ± 5.3	26 weeks	200 mg/d RSV		Placebo	Auditory Verbal Learning Test		+
Brickman et al. (2014)	*N* = 37	Healthy 50–69 y	3 months	High cocoa flavonol-diet		Low flavonol-diet	ModBent task		+

*NP, Neuropsychological; P, Age of placebo group; RSV, resveratrol; I, Age of intervention group; y, years of age; +, positive effect on cognition; ○, no effect on cognition*.

Both RSV and flavonols could contribute to a better cognitive performance due to their protective effects against oxidative stress, which increases with age and is a risk factor for age-associated cognitive decline. Further possible neuroprotective mechanisms of polyphenols including RSV are reduced mitochondrial dysfunction, glucose toxicity, oxidative damage, and chronic inflammation, by improving glucose metabolism and vascular functions and by activating so-called longevity genes including the sirtuins. For further discussions see e.g., Calabrese et al. (2008, 2009), Sun et al. (2011), Crichton et al. (2013) and Witte et al. (2014).

## Conclusion and Outlook

A majority of available studies on the topic suggest that consumption of LC-n3-FA with fish or fish oil-supplements and plant polyphenols such as flavonols and RSV exerts positive effects on brain health and cognition in older humans. However, with regard to LC-n3-FA supplementation using fish oil, a final recommendation based on RCTs cannot be drawn, as some studies could not detect a positive effect. Here, more large-scale RCTs that, for example, also control for other fatty acid intake are needed to support a significant benefit of regular supplementary LC-n3-FA intake in maintaining cognitive performance. Considering polyphenols, the evidence based on high-quality RCTs is even less clear, given that only few reliable studies are available to date with different formulas and different duration of the intervention. Yet, those few studies were promising, and the animal literature provided convincing examples that polyphenols are highly potent in activating possible neuroprotective pathways, warranting the initiation of large-scale RCTs in humans on supplementary flavonol or RSV. Moreover, attempts to study in parallel the underlying mechanisms in humans, e.g., using high-resolution MRI, are especially important to further strengthen possible hypotheses that are mainly based on animal research. Future studies also need to address whether intervention-induced changes in LC-n3-FA or polyphenol intake relate to changes in fatty acid or polyphenol content at the brain level in humans, e.g., using post-mortem techniques.

Besides that, additive or synergistic effects between single dietary components come increasingly into focus. Diet is more than the sum of its components, which is considered in the concept of “food synergy”. The assumption is that interactions and synergistic effects of the single food components occur as they are consumed in the framework of a balanced diet (Jacobs et al., 2009). For example, antioxidant nutrients can protect LC-n3-FA from peroxidation to which they are particularly susceptible due to their multiple double bounds (Barberger-Gateau, 2014). Also, even though studies on single nutrients and their interactions might help to explain the beneficial effects of dietary patterns, there is an even greater framework. Yannakoulia et al. (2015) propose not only the additive and synergistic effects of single nutrients or foods, but also add other lifestyle behaviors like physical activity, social support, sharing food, having lengthy meals and post-lunch siestas to that explanatory approach. Regardless of all the modernization processes happening (Bach-Faig et al., 2011), the lifestyle of the Mediterranean countries remains an UNESCO World Cultural Heritage and could thus contribute to a multitude of insights regarding brain functioning and healthy aging (Bach-Faig et al., 2011). First publications of large-scale RCTs, such as Valls-Pedret et al. (2015) and Ngandu et al. (2015), provide a strong level of scientific evidence for the beneficial effects of the MeDi on cognitive functions. In addition, ongoing multidomain interventional trials like the Finnish Geriatric Intervention Study to Prevent Cognitive Impairment and Disability (FINGER) will help to gain further insights into the beneficial effects of the MeDi-lifestyle and its components on cognition and brain function. The FINGER-study is a multi-center RCT and includes nutritional guidance, regular exercise, cognitive training and social activity, as well as management of metabolic and vascular risk factors, and might thus shed comprehensively further light on possible mechanisms of how modifiable lifestyle factors could help to maintain cognitive functions throughout age (Kivipelto et al., 2013).

Summing up, LC-n3-FA and polyphenols such as RSV are highly investigated substances in the framework of the MeDi. Even though, more studies are needed to clarify the main effects and their underlying mechanisms, they seem to be promising with regard to their impact on brain structure and function in aging.

## Conflict of Interest Statement

The authors declare that the research was conducted in the absence of any commercial or financial relationships that could be construed as a potential conflict of interest.
